# Heteroplasmic Variants of Mitochondrial DNA in Atherosclerotic Lesions of Human Aortic Intima

**DOI:** 10.3390/biom9090455

**Published:** 2019-09-06

**Authors:** Igor A. Sobenin, Andrey V. Zhelankin, Zukhra B. Khasanova, Vasily V. Sinyov, Lyudmila V. Medvedeva, Maria O. Sagaidak, Vsevolod J. Makeev, Kira I. Kolmychkova, Anna S. Smirnova, Vasily N. Sukhorukov, Anton Y. Postnov, Andrey V. Grechko, Alexander N. Orekhov

**Affiliations:** 1 Institute of Experimental Cardiology, National Medical Research Center of Cardiology, 121552 Moscow, Russia (Z.B.K.) (V.V.S.) (V.N.S.) (A.Y.P.); 2 Institute of General Pathology and Pathophysiology, 125315 Moscow, Russia; 3 Research Institute of Threpsology and Healthy Longevity, Plekhanov Russian University of Economics, 115093 Moscow, Russia; 4 Federal Research and Clinical Center of Physical-Chemical Medicine, 119435 Moscow, Russia; 5 Federal Research Center of Transplantology and Artificial Organs, 123182 Moscow, Russia; 6 Vavilov Institute of General Genetics, 117971 Moscow, Russia (M.O.S.) (V.J.M.); 7 Moscow Institute of Physics and Technology, Dolgoprudny, 141701 Moscow Region, Russia; 8 Engelhardt Institute of Molecular Biology, 119991 Moscow, Russia; 9 Institute for Atherosclerosis Research, Skolkovo Innovation Center, 143026 Moscow, Russia (K.I.K.) (A.S.S.) (A.N.O.); 10 Research Institute of Human Morphology, 117418 Moscow, Russia; 11 Federal Scientific Clinical Center for Resuscitation and Rehabilitation, 141534 Moscow Region, Russia

**Keywords:** atherosclerosis, mitochondrial DNA mutations, next generation sequencing, mitochondrial DNA copy number, unaffected intima, fatty infiltration, fatty streaks, lipofibrous plaque, fibrous plaque

## Abstract

Mitochondrial dysfunction and oxidative stress are likely involved in atherogenesis. Since the mitochondrial genome variation can alter functional activity of cells, it is necessary to assess the presence in atherosclerotic lesions of mitochondrial DNA (mtDNA) heteroplasmic mutations known to be associated with different pathological processes and ageing. In this study, mtDNA heteroplasmy and copy number (mtCN) were evaluated in the autopsy-derived samples of aortic intima differing by the type of atherosclerotic lesions. To detect mtDNA heteroplasmic variants, next generation sequencing was used, and mtCN measurement was performed by qPCR. It was shown that mtDNA heteroplasmic mutations are characteristic for particular areas of intimal tissue; in 83 intimal samples 55 heteroplasmic variants were found; mean minor allele frequencies level accounted for 0.09, with 12% mean heteroplasmy level. The mtCN variance measured in adjacent areas of intima was high, but atherosclerotic lesions and unaffected intima did not differ significantly in mtCN values. Basing on the ratio of minor and major nucleotide mtDNA variants, we can conclude that there exists the increase in the number of heteroplasmic mtDNA variants, which corresponds to the extent of atherosclerotic morphologic phenotype.

## 1. Introduction

The emergence and development of atherosclerotic lesions in the arterial wall are known to occur locally and even focally. The focality of atherosclerotic lesions can be explained by the differences in hemodynamic stress in different parts of the vessel, the local changes in the permeability of the vascular endothelium, and the presence of morphologically and functionally distinct subpopulations of cells able to form local clusters under the endothelial lining [1,2,3]. The alterations in the structure of the mitochondria, mitochondrial dysfunction, and oxidative stress are frequently observed in atherosclerotic lesions [4,5]. Reactive oxygen species (ROS) generated by mitochondria are involved in processes that contribute to the progression of atherosclerotic lesions [6]. Oxidative stress in mitochondria is considered to be the cause of increased mutation rate in mitochondrial DNA (mtDNA), that is 6–17 times higher compared to the nuclear DNA. The presence of multiple copies of mtDNA in single cell explains the phenomenon of mtDNA heteroplasmy, i.e., the variable proportion of normal and mutant mtDNA copies within the cell or tissue [7,8,9]. Recent studies have confirmed the presence of mtDNA heteroplasmy in various types of human tissues; there are heteroplasmic mutations that are accumulated with ageing [10]. The assessment of mtDNA heteroplasmic mutation load, as well as mtDNA copy number (mtCN) is a plausible way to explain the focality of atherosclerotic lesions [11,12,13,14]. Heteroplasmic mtDNA mutations are known to be associated with different age-related diseases [7,15,16,17]. The presence of mtDNA heteroplasmy, which is conventionally regarded as the molecular basis for mitochondrial cytopathies, have been found also in healthy individuals without any symptoms of such diseases [18]. For a number of heteroplasmic mtDNA mutations, the accumulation of mutant mtDNA copies in specific tissues have been proved [10]. Possibly, heteroplasmic mtDNA mutations are of somatic origin, since the activity of mitochondrial function and ROS production vary significantly between different types of tissues [19]; however, the significant part of heteroplasmic mtDNA mutations, or variants, may be inherited by maternal line [20,21]. Pathogenic mtDNA mutations affect respiratory chain functioning, usually forming the mosaic structure of its deficiency. Cells possessing the level of heteroplasmy exceeding a certain threshold, exhibit mitochondrial dysfunction, whereas in the adjacent cells with lower mutational load the respiratory chain remains fully functional [4,8,22,23,24]. A similar pattern can be observed in atherosclerosis: the normal (unaffected) areas of arterial wall exist side by side with different types of atherosclerotic lesions, and mtDNA mutational load exhibits mosaic structure, as well [25,26]. The recent studies based on the use of high-throughput sequencing technologies have demonstrated that mtDNA heteroplasmy occurs commonly and usually has the frequency rate of about 2% [27,28]. This study was undertaken to test the hypothesis that the frequency rate of mtDNA heteroplasmic mutations increases in atherosclerotic lesions, thus providing the mechanistic explanation of atherosclerosis focal progression.

## 2. Materials and Method

### 2.1. Human Aortic Intimal Tissue Autopsy Samples

A total of 7 autopsy samples of thoracic aorta were taken within 48 post-mortem hours from 7 different subjects. The study protocol was approved by the Ethics Committee of the Institute for Atherosclerosis Research, Moscow (protocol No. 7 of 17 October 2017). Gender, age and pathological diagnosis of subjects are shown in Table 1. 

The vessels were opened longitudinally and washed with ice-cold phosphate buffered saline, pH = 7.6. Each section contained normal (unaffected) areas of aortic intima, as well as 5 different types of atherosclerotic lesions: Fatty infiltrations (type I lesions, FI), fatty streaks (type II lesions, FS), lipofibrous plaques (type Va lesions, LFP) and fibrous plaques (type Vc lesions, FP). To determine the type of lesion, the fragments of aortic intima were examined macroscopically and were classified in accordance with the classification of the Atherosclerosis Council of the American Heart Association [29,30]. Aortic intima was mechanically separated from medial layer, and 11–12 samples of intimal tissue sizing up to 0.25 cm^2^ were carved from each autopsy sample. Additionally, the tissue samples from skeletal muscle (SM), myocardial muscle (MM), liver (LIV), and spleen (SPL) were taken at each autopsy, except for the case as_01. Thus, a total of 83 aortic intimal samples and 24 samples from other tissues were collected for the study and kept at −80 °C prior to DNA extraction. 

### 2.2. DNA Extraction and mtDNA Enrichment

Each tissue sample was homogenized in liquid nitrogen, and total genomic DNA was extracted from 50 mg of sample using Qiagen DNEasy kit (QIAGEN, Germantown, MD, USA). Enrichment of mtDNA was carried out by long-range PCR with high-fidelity Q5 polymerase (New England Biolabs, Ipswich, MA, USA) and two specific primer sets (Set 1: ACGGGCTCACATCACCCCATAA and GTACGGCCAGGGCTATTGGT; Set 2: ACAACTAACCTCCTCGGACTCCT and CVTGGCTGGCACGAAATTGACC), with amplification of two overlapping mtDNA fragments with the length of 8378 and 8702 b.p., respectively; the overlappings were at positions 623-893 (covers the end of tRNA-phenylalanine coding sequence, minor H-strand promoter, the beginning of 12S RNA coding sequence), and at positions 8767–9001 (covers the part of ATP synthase F0 subunit 6 coding sequence). Amplification included 33 cycles and was set up in accordance with recommendations on the use of Q5 polymerase. The lengths of obtained fragments were confirmed by electrophoresis of the whole sample in 1% agarose gel. Target large fragments (bands in 8–10 kB region) were cleaned up from PCR mix components by elution from agarose gel on columns using specific elution kit (Evrogen, Moscow, Russia). Concentration of DNA fragments was measured by spectrophotometry using NanoDrop nanophotometer (Implen, München, Germany), and both of mtDNA-enriched fragments were pooled for each sample in equal weight amounts in single tube.

### 2.3. Whole mtDNA Sequencing

Roche 454 GS Junior Titanium System was used for high-throughput sequencing of mtDNA. Five hundred nanogram of the enriched mtDNA fraction was taken for DNA libraries preparation and further sequencing. «Shotgun» DNA libraries were made by fragmentation of amplicons with nitrogen under pressure of 2.1 bar and ligation of specific sequencing adapters (GS MID Adaptors Kit, Roche Applied Science, Madison, WI, USA). Sequencing workflow was performed according to the manufacturer’s recommendations using appropriate instruments and reagents. The following parameters were achieved: mean reading length, 458 bp; the mean number of readings, 18,734; the mean number of nucleotides read in one sample, 8.65 million b.p. The mean percent of mapped readings accounted for 93%. To detect heteroplasmic variants of mtDNA, sequences with more than 300-fold coverage of the mitochondrial genome were taken, thus allowing reliable detection of variants with a level of heteroplasmy of at least 1% in the presence of direct and reverse readings of the mutant allele.

### 2.4. Measurement of mtDNA Copy Number

MtDNA copy number (mtCN) was measured by qPCR with three primer sets according to Venegas and Halberg [31] by comparative ct (2^−ΔΔ*C*T^) method [32], using SYBR Green Supermix (Evrogen, Moscow, Russia) and BioRad CFX96 Touch Real-Time PCR Detection System (BioRad, Hercules, CA, USA). Total genomic DNA extracted from tissue samples was taken in amount of 25 ng per 25 μL reaction volume. Variations between 3 replicative measurements did not exceed 0.5 cycles. MtCN was calculated as a ratio between mtDNA and nucDNA copies, with the formula 2^(−∆Cq)^, where ∆Cq = Cq (nDNA set) − Cq_mean_ (mtDNA set; mtDNA alternative set). 

### 2.5. Bioinformatical and Statistical Analysis of Sequencing Data

Since the use of 454 technology for sequencing is characterized by limitations when sequencing homopolymers, the processing of the data should be performed with accurate handling and filtration of reads avoiding artefacts in heteroplasmy detection [33]. Therefore, 5′-adaptor sequences were cut from the reads using Cutadapt software [34]. Filtration by quality was performed with Trimmomatic [35]. Reads shorter than 30 b.p. were eliminated with the quality threshold of 20 (SLIDINGWINDOW option with window size 3, required quality 20, minimal read length 30). Then, the reads were mapped on *Homo sapiens* mitochondrion complete genome sequence (NCBI Reference Sequence, GenBank accession number NC_012920.1) [36] with the use of BWA-MEM software [37]. To avoid simultaneously mapping of reads in different places, reads with MAPQ < 50 were excluded from further consideration. SNPs were obtained using Bcf-tools and Samtools, and annotated in Annovar software [38,39].

Extraction of heteroplasmic SNPs from the general SNP pool was performed with the following criteria:

- both of two variant nucleotides at the SNP site should be presented in the reads mapped to rCRS;
- the coverage of heteroplasmic site should be within the limit of 0.5–2.0-fold of the mean coverage depth;
- SNPs on homopolymeric sites were excluded from further analysis;
- heteroplasmic site should be covered with forward and reverse reads.

Further, heteroplasmies which did not satisfy the last condition, but were found in the same tissue samples of the same individual and in the same position, were additionally analyzed. For minor allele frequencies (MAFs) of such a pair of heteroplasmies binominal test was performed. If p-value exceeded 0.95, the heteroplasmy was considered assuming that in the same type of lesion heteroplasmies can match and its detection is not a sequencing error. MAFs for heteroplasmic variants were calculated as a ratio of the number of minor nucleotide variant and the total number of nucleotides in the particular position of mtDNA. Wilcoxon signed-rank test was used to obtain values of significance between mean MAFs of the heteroplasmies between individuals and between unaffected aortic tissue and early changes (N and FI) and evident atherosclerotic lesions (FS, LFP, and FP). Chi-squared test was used for verification of the total number of heteroplasmies in unaffected and atherosclerotic tissue.

The results were considered as significant with the criterion of *p*-value < 0.05. Diagram and plot creation, statistical analysis and data processing were performed with SciPy [40], pandas [41], Matplotlib [42], and seaborn [43] software. MtDNA mutations were named according to HGVS nomenclature [44]. RefSeq NC_012920.1 prefix was used in the full names of all mutations.

## 3. Results

### 3.1. Heteroplasmic Variants of mtDNA

**Figure 1:**
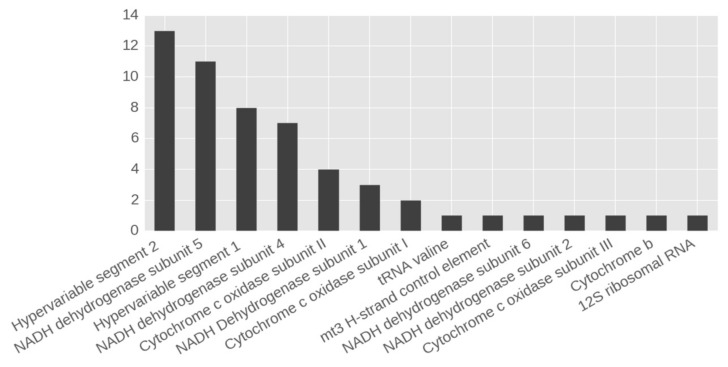
The number of heteroplasmic variants found in regions and genes of mitochondrial DNA (mtDNA).

**Figure 2:**
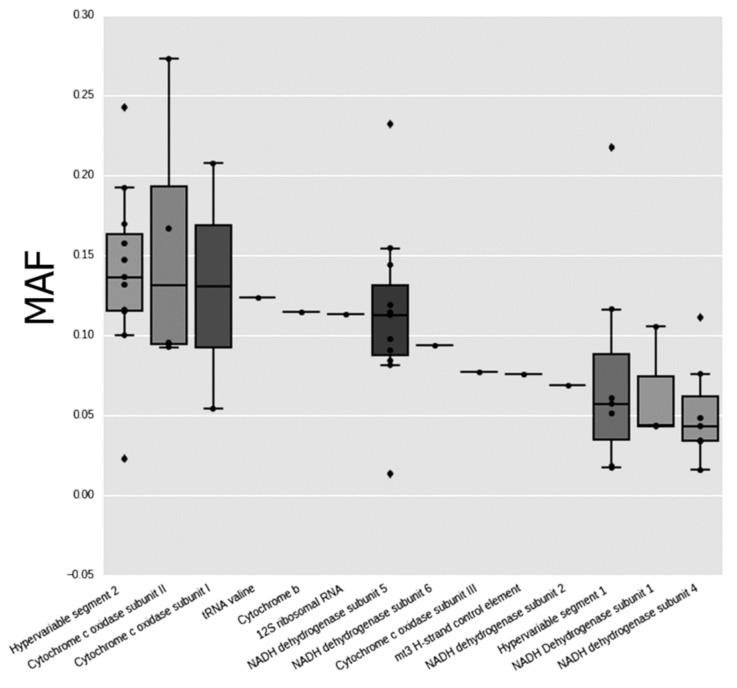
The boxplot showing minor allele frequencies (MAF) distribution of heteroplasmic variants in regions and genes of mtDNA. Circles, the cases falling into observed distribution; diamonds, outliners.

The total of 55 cases of heteroplasmy that satisfied all required criteria of selection were found in 27 mtDNA positions, with more than 90% of all sequencing reads being mapped on *Homo sapiens* mitochondrion complete genome sequence. Heteroplasmic variants were found in all male and female individuals analyzed. Distribution of the number and frequency of heteroplasmies by mtDNA genes are shown in Figure 1 and Figure 2. 

**Figure 3:**
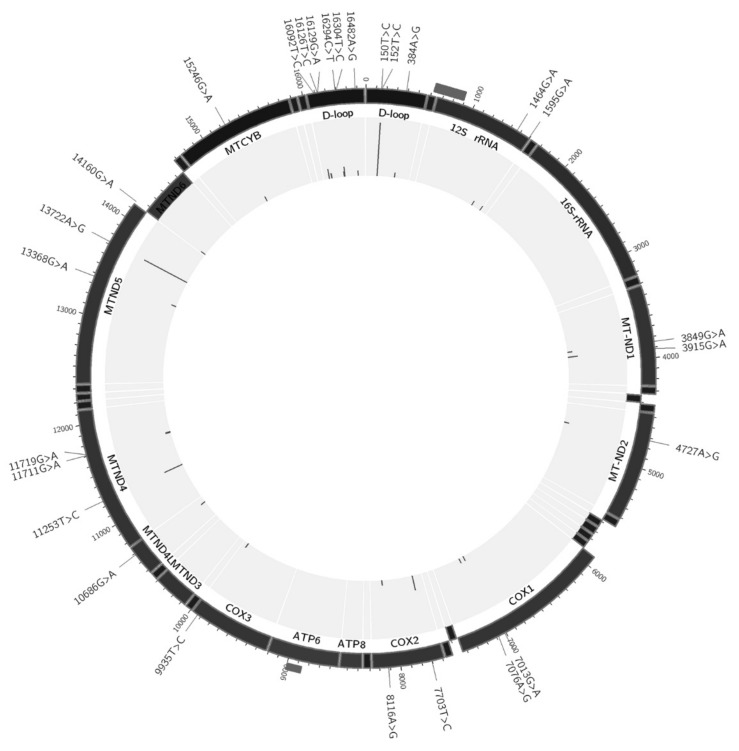
MtDNA map of heteroplasmic variants found in the study. Dark grey rectangles outside mark overlapping mtDNA fragments during mtDNA enrichment at positions 623–893 and 8767–9001; none of detected variants were located within these ranges.

The most cases of heteroplasmy were found in hypervariable segment (HVS) and in NADH-dehydrogenase subunits 4 and 5. Interestingly, only 2 heteroplasmic variants were found in different individuals (m.152T > C in 3 cases, and m.16304T > C in 2 cases), and both these variants were located in HVS. All other heteroplasmic variants were unique for each case. The final set of all heteroplasmic variants and their location in mtDNA are shown in Figure 3. This information is provided also in Table 2 and Table S1 (Supplementary Materials).

**Figure 4:**
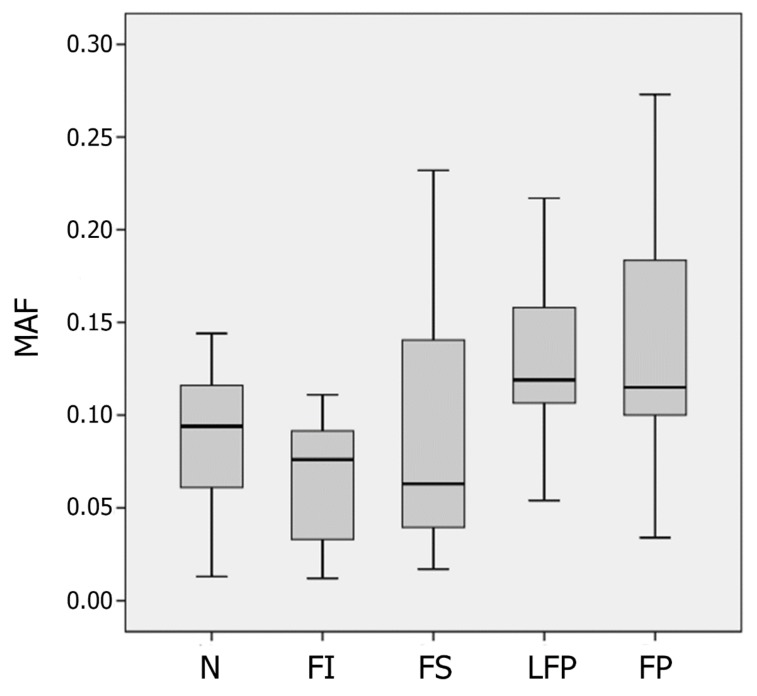
The boxplot showing the mean minor allele frequencies (MAF) values of heteroplasmic mtDNA variants in unaffected aortic intima (N) and different types of atherosclerotic lesions (FI, fatty infiltration; FS, fatty streaks; LFP, lipofibrous plaque; FP, fibrous plaque).

The mean MAF values of heteroplasmic variants were growing from unaffected aortic tissue to fibrous and lipofibrous plaques (Figure 4). The chi-square test of independence was performed for the contingency table (Table 3). 

The resulting *p*-value was 2.72 × 10^−10^, what meant that the hypothesis of MAF independence from the lesion type was to be rejected. Wilcoxon signed-rank test was performed for the data given in Table 4, and *p*-value was 0.018, thus demonstrating the significance of MAF shift from unaffected tissue samples to atherosclerotic lesions.

### 3.2. MtDNA Copy Number

**Figure 5:**
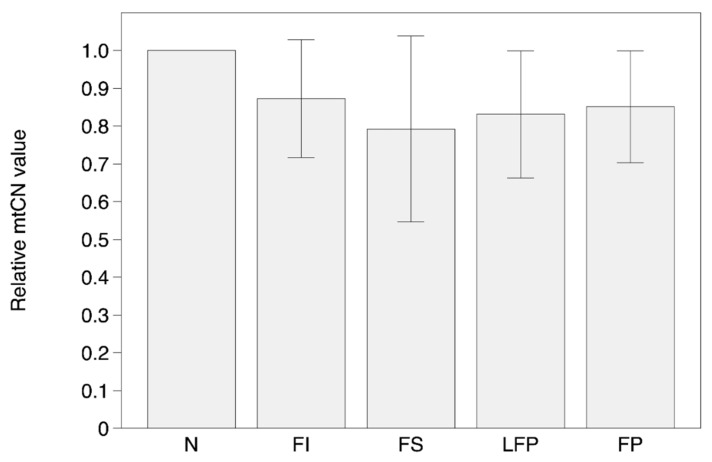
The relative mtCN values in unaffected aortic intima (N) and different types of atherosclerotic lesions (FI, fatty infiltration; FS, fatty streaks; LFP, lipofibrous plaque; FP, fibrous plaque).

**Figure 6:**
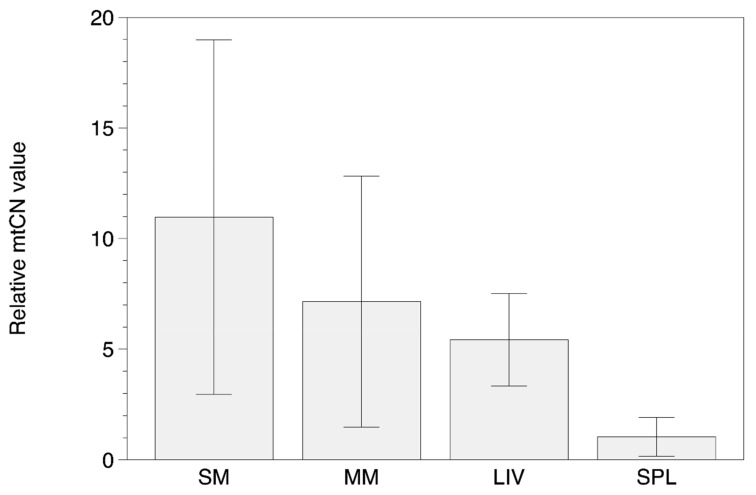
The relative MtDNA copy number (mtCN) values in tissue samples from skeletal muscle (SM), myocardial muscle (MM), liver (LIV) and spleen (SPL).

Results of mtDNA copy number (mtCN) measurements are presented in Table S2 (Supplementary Materials) and Figure 5 and Figure 6. The data were normalized to an arbitrary sample (unaffected intima) in each case to provide a fold difference between samples. 

In general, there were no significant differences in the mtCNs in the different types of atherosclerotic lesions of aortic intima. The overall mtCN distribution between N, FI, FS, FP, and LFP samples was similar among the majority of individuals: the MTCN values ranged 200–250 with CV no more than 25% (Table S2 (Supplementary Materials), Figure 5). However, one sample (as_01) have demonstrated reduced mtCN values in aortic tissue, and one more sample (as_04)—the elevated values. The results of mtCN determination in another types of tissues showed clear differences in mean mtCN values with maximum in SM and minimum in SPL samples (Table S2 (Supplementary Materials), Figure 6).

## 4. Discussion

Our present findings showed that the presence of mtDNA heteroplasmic variants in unaffected and atherosclerotic intima of human aorta is a common phenomenon but lacking the obvious tendency. At least, none of the detected mtDNA variants could be described as lesion-specific one. Previously, several mtDNA heteroplasmic variants were reported to be associated with atherosclerotic lesions [25,26,45]. It is notable that pyrosequencing with short reads was mainly applied within those studies. Using another technique of amplification and sequencing, these variants were either not found in the similar types of samples, ore were filtered out at the stage of raw data analysis. Following the results of 454-sequencing, it may be assumed that mtDNA heteroplasmy can be found presumably in individual cases. Mean coverage of all heteroplasmic sites in our study was 80.3 ± 57.2; the mean MAF accounted for 0.09, with 12% mean heteroplasmy level. The half of the observed cases of heteroplasmy had the coverage of more than 60 reads, and for these cases the lowest detectable heteroplasmy level accounted for 3%, since the minimum of 2 reads (forward and reverse ones) was required for detection. Therefore, it may be assumed that the mtDNA variants with heteroplasmy level of 10–20% would likely have been detected by 454-sequencing. A lack of compatibility between different studies is a challenging question that can be possibly solved by several explanations. There can be a bias in the amplification step of short reads in pyrosequencing method leading to the increase of detectable values of heteroplasmy level of mtDNA mutations that remain undetectable using 454-sequencing method. Furthermore, in short read pyrosequencing clean-up of target PCR-product after the amplification step is not performed, and erroneous heteroplasmy levels can possibly be detected if there is a presence of nuclear mtDNA pseudogenes (NUMTS) amplicons with the same size in the amplicon pool. With the use of long-range PCR prior to sequencing, target large mtDNA amplicons can be cleaned up from possibly occurring NUMTS short PCR products. 

MtCN is now increasingly studied as a possible biomarker of aging and disease [46,47,48]. A number of studies were carried out to reveal the role of mtCN in atherosclerotic-related diseases and diabetes [11,12,13,14]. Chien et al. [49] have measured mtCN in human blood vessel tissue and have found that diabetic patients had significantly fewer copies of mtDNA than non-diabetic subjects, and patients with arterial stenosis had fewer copies compared to those without arterial stenosis. In our study we did not compare mtCN in patients with different severity of atherosclerosis, but our aim was to study the differences in mtCN relative values between different types of lesions in the same vessel. There was no significant increase or decrease in mtCN, which could correspond to atherosclerosis progression from unaffected tissue to advanced atherosclerotic lesions. Although there were differences in mtCN distribution between different subjects, the most common distribution seemed to be without significant differences in mtCN of different lesion types. Some samples demonstrated lowered mtCN relative values in lesions compared to normal tissue, but there was no regularity of this trend in the general sample. Relying on these findings we can conclude that there is no exact evidence of increased mitochondria biogenesis as well as increased mitophagy or mitochondria elimination in atherosclerotic lesions compared to unaffected intima. MtCN of aortic intimal tissue was less than that in myocardial or skeletal muscle and liver tissue. Our results of mtCN measurements in different types of human tissues (SM, MM, LIV, and SPL) are well consistent with previously published data obtained by both qPCR and high-throughput sequencing [50,51].

Comparing our results with the massively parallel sequencing data on assessment of heteroplasmy across ten tissues obtained by Samuels at al. in 2013 [51], we found one match in heteroplasmic mutation (m.16126T > C) found in kidney and skeletal muscle with the quite similar MAF). Also, studying pathologically changed tissues, we have found more heteroplasmic variants in the coding region of mtDNA, and some of them were missence mutations, located in cytochrome c oxidase and NADH-dehydrogenase subunits and cytochrome b genes. Some of these mutations (m.10686G > A, m.11253T>C, m.14160G > A, and m.11711G > A) were previously described [52,53,54,55,56,57], but two variants were novel (m.7703T > C and m.15246G > A). Interestingly, some of these missence heteroplasmic mutations were found in cancer tissues, what leads to suggestion of somatic nature of these mutations as a cause of changes in tissue microenviroment. 

This study has several limitations. First, since the composition of cell types in human aortic intima is different in unaffected and atherosclerotic samples, it may be reliable to assume the influence on mtDNA copy number and mutational load. Earlier we have characterized the cell composition in unaffected and atherosclerotic intima (initial lesions, fatty streaks, lipofibrous plaques and fibrous plaques [58,59]. It was shown that all types of intimal samples contain smooth muscle cells containing smooth muscle α-actin (approximately 50% of total cell population), the cells of hematogenic origin (lymphocytes and macrophages, from 3% to 20% of total cell population), and pericyte-like cells (from 30% to 40% of total cell population). More, pericyte-like cells in unaffected and atherosclerotic aortic intima are characterized by lowered proportion of resting cells in initial lesions and fatty streaks, and the increased proportion of activated cells in lipofibrous and fibrous plaques [60]. One should also consider the fact of simultaneous expression of macrophage antigen by subendothelial smooth muscle cells [61]. Such characteristics of cellular composition, along with technical problems of isolation of alive specific cell types from subendothelial intima for further DNA isolation, make the estimation of their influence on mtDNA copy number and mutational load a real challenge. Next, the comparative ct method used for mtCN estimation does not allow for absolute copy number count like standard curve method; all possible mtDNA deletions could not necessarily be caught with next generation sequencing depending on the criteria used to throw out reads. Finally, the sample size was small (all intimal samples were obtained from 7 autopsy cases, 4 males and 3 females), it was impossible to make statistically valid estimates of the distribution of heteroplasmic variants across individuals, of the difference between male and female individuals, on the overlap between heteroplasmic variants and known haplogroup motifs, on the association between age and mtDNA variants, etc. Although the same heteroplasmic variants were usually found in the replicates of the same types of atherosclerotic lesions within the same autopsy sample, statistical estimate of conservation was impossible as well, since there was maximum of 2–3 replicates per specific sample.

## 5. Conclusions

The main finding in this study was the progressive increase in the number of occurring heteroplasmic mtDNA variants from normal (non-atherosclerotic) intima to advanced lesions, like lipofibrous and fibrous plaques. Therefore, the possibility of somatic origin of mtDNA heteroplasmic variants due to the increased ROS production and impaired mitochondrial function supports the hypothesis of accumulation of mutant mtDNA copies during atherogenesis. Basing on the ratio of minor and major nucleotide mtDNA variants, we can conclude that there exists the increase in the number of heteroplasmic mtDNA variants, which corresponds to the extent of atherosclerotic morphologic phenotype. The accumulating global data on the mtDNA damage in atherosclerosis [62,63] make this hypothesis plausible, taking in account the role of increased oxidative stress as the mechanistic factor of atherosclerotic risk. However, oxidative stress cannot be considered as the main mechanism of origination of mtDNA somatic mutations. The results of the recent studies argue against oxidative damage hypothesis [64,65], and suggest, for example, that replication errors by DNA polymerase γ and/or spontaneous base hydrolysis may be more responsible for accumulation of mtDNA point mutations [66]. The nature of pathogenic and mechanistic role of heteroplasmic mtDNA variants in the development of disease phenotype is far from understanding. It is even not possible to consider them as the causation or the consequence of pathology. To answer this fundamental question, it is necessary to study the functional consequences of mitochondrial site-specific DNA damage. The use of cell-based models seems to be a promising way; for example, cytoplasmic hybrids (cybrids) are currently among the best for the study of mitochondrial dysfunction, although this model has certain disadvantages [67]. Another and possibly better approach is aimed to the direct editing of mtDNA for the precise studies of the effects of single nucleotide substitution [68]; however, direct modification of the mitochondrial genome is not yet technically solved scientific and technical problem, because there is still no reliable way of delivering nucleic acids and molecular structures to the mitochondria of living cells [69,70]. In any way, the disclosure of associations between mtDNA damage and pathology will provide new knowledge of atherogenesis as the age-related degenerative disease process and build the basis for developing new approaches to diagnosis, prevention and treatment. 

## Figures and Tables

**Table 1:** Pathologic characteristics of aortic intima autopsy samples.

Case No. (ID)	Age	Gender	Pathologic Diagnosis	The Number of Tissue Samples				
				N	FI	FS	LFP	FP
as_01	85	f	macrofocal atherosclerosis	2	1	2	3	3
as_02	83	m	pulmonary heart disease, thromboembolia of small pulmonary arteries, macrofocal cardiosclerosis	2	2	2	3	3
as_03	86	f	pulmonary heart disease, bilateral confluent bronchopneumonia, diffuse microfocal cardiosclerosis	2	2	2	3	3
as_04	87	f	pulmonary artery thromboembolia, macrofocal cardiosclerosis	2	3	3	2	2
as_05	60	m	gastrorrhagia	3	2	2	2	3
as_06	83	m	diffuse microfocal cardiosclerosis, cardiohepatic insufficiency, right-focal abscessed confluent pneumonia	2	2	2	3	3
as_07	83	m	right kidney cancer, cancerous cachexia, macrofocal atherosclerosis	2	3	2	2	3
Total number of samples				15	15	15	18	20

m—male; f—female; N—normal (unaffected) intima; FI—fatty infiltration; FS—fatty streak; LFP—lipofibrous plaque; FP—fibrous plaque.

**Table 2:** Heteroplasmic mtDNA variants found in the aortic intimal tissue.

Position	Gene/Region	Nucleotide Change	Mutation Type (for Protein-Coding Genes) and Aminoacid Change	Total Number of Hetero-Plasmy Cases	Mean MAF	SD
150	Hypervariable segment 2	C > T	---	2	0.0175	0.0078
152	Hypervariable segment 2	T > C	---	11	0.1528	0.0407
384	mt3 H-strand control element	A > G	---	1	0.0760	
1464	12S ribosomal RNA	G > A	---	1	0.1130	
1595	tRNA valine	G > A	---	1	0.1240	
3849	NADH Dehydrogenase subunit 1	G > A	syn:L-L	1	0.1050	
3915	NADH Dehydrogenase subunit 1	G > A	syn:G-G	2	0.0430	0.0000
4727	NADH dehydrogenase subunit 2	A > G	syn:M-M	1	0.0690	
7013	Cytochrome c oxidase subunit I	G > A	syn:T-T	1	0.0540	
7076	Cytochrome c oxidase subunit I	A > G	syn:G-G	1	0.2070	
7703	Cytochrome c oxidase subunit II	T > C	non-syn:Y-H	3	0.1183	0.0422
8116	Cytochrome c oxidase subunit II	A > G	syn:G-G	1	0.2730	
9935	Cytochrome c oxidase subunit III	T > C	syn:H-H	1	0.0770	
10686	NADH dehydrogenase subunit 4	G > A	non-syn:V-M	1	0.0760	
11253	NADH dehydrogenase subunit 4	T > C	non-syn:I-T	4	0.0398	0.0069
11711	NADH dehydrogenase subunit 4	G > A	non-syn:A-T	1	0.1110	
11719	NADH dehydrogenase subunit 4	G > A	syn:G-G	1	0.0160	
13368	NADH dehydrogenase subunit 5	G > A	syn:G-G	1	0.0130	
13722	NADH dehydrogenase subunit 5	A > G	syn:L-L	10	0.1229	0.0453
14160	NADH dehydrogenase subunit 6	G > A	non-syn:R-W	1	0.0940	
15246	Cytochrome b	G > A	non-syn:G-D	1	0.1140	
16092	Hypervariable segment 1	T > C	---	2	0.0410	0.0141
16126	Hypervariable segment 1	T > C	---	1	0.0170	
16129	Hypervariable segment 1	G > A	---	1	0.2170	
16294	Hypervariable segment 1	C > T	---	1	0.1160	
16304	Hypervariable segment 1	T > C	---	2	0.0395	0.0304
16482	Hypervariable segment 1	A > G	---	1	0.0570	

syn—synonymous mutation; non-syn—non-synonymous mutation.

**Table 3:** Contingency table for minor and major variants in heteroplasmic mutations.

Parameter	The Type of Atherosclerotic Lesion				
	N	FI	FS	LFP	FP
The number of minor variants	68	70	106	113	134
The number of major variants	786	1049	965	732	801

N—normal (unaffected) intima; FI—fatty infiltration; FS—fatty streak; LFP—lipofibrous plaque; FP—fibrous plaque.

**Table 4:** Mean minor allele frequencies (MAF) values in unaffected (N) and atherosclerotic (FI, fatty infiltration; FS, fatty streaks; LFP, lipofibrous plaque; FP, fibrous plaque) aortic tissue samples.

Case No. (ID)	Mean MAF	
	N+FI	FS+FP+LFP
as_01	0	0.115
as_02	0.097	0.107
as_03	0.074	0.133
as_04	0.060	0.111
as_05	0.098	0.136
as_06	0.039	0.055
as_07	0.019	0.211

## Supplementary Materials

The following are available online at https://www.mdpi.com/2218-273X/9/9/455/s1, Table S1: MAF values for heteroplasmic mtDNA variants found in the aortic intimal tissue, Table S2: Relative mtCN values in the observed tissue samples.
