# Supplementary material for: Heteroplasmic Variants of Mitochondrial DNA in Atherosclerotic Lesions of Human Aortic Intima
Source: Biomolecules. 2019 Sep 6;9(9):455. doi: 10.3390/biom9090455 (PMC6770808; doi:10.3390/biom9090455)
Supplement: Supplementary file 1 [file biomolecules-09-00455-s001.zip › S1_Table.pdf]

Table S1: MAF values for heteroplasmic mtDNA variants found in the aortic intimal tissue.

Sample name	Sample type	Position in mtDNA	MAF
as_1	FS	16092	0.031
as_1	FS	16092	0.051
as_1	FS	1595	0.124
as_1	N	14160	0.094
as_1	FP	8116	0.273
as_2	FI	11711	0.111
as_2	FI	7703	0.093
as_2	FS	7703	0.167
as_2	LFP	7013	0.054
as_2	LFP	152	0.1
as_2	FP	7703	0.095
as_3	N	152	0.132
as_3	N	152	0.116
as_3	N	13368	0.013
as_3	FS	16126	0.017
as_3	FS	152	0.157
as_3	FS	16304	0.018
as_3	LFP	152	0.169
as_3	LFP	152	0.136
as_3	LFP	152	0.115
as_3	FP	152	0.242
as_3	FP	152	0.192
as_3	FP	15246	0.114
as_3	FP	3849	0.105
as_3	FP	152	0.175
as_4	N	16304	0.061
as_4	LFP	10686	0.076
as_4	LFP	152	0.147
as_5	N	13722	0.144
as_5	N	13722	0.113
as_5	N	9935	0.077
as_5	FI	13722	0.081
as_5	FI	384	0.076
as_5	FI	13722	0.09
as_5	FI	13722	0.098
as_5	FS	13722	0.084
as_5	FS	13722	0.232
as_5	LFP	13722	0.119
as_5	FP	13722	0.114
as_5	FP	16294	0.116
as_5	FP	13722	0.154
as_6	N	11253	0.034
as_6	FI	11253	0.043
as_6	FI	3915	0.043
as_6	FS	16482	0.057
as_6	FS	11253	0.048
as_6	FS	4727	0.069
as_6	LFP	1464	0.113
as_6	FP	3915	0.043
as_6	FP	11253	0.034
as_7	FI	150	0.012
as_7	FI	11719	0.016
as_7	FI	150	0.023
as_7	LFP	7076	0.207
as_7	LFP	16129	0.217
