# Supplementary material for: Heteroplasmic Variants of Mitochondrial DNA in Atherosclerotic Lesions of Human Aortic Intima
Source: Biomolecules. 2019 Sep 6;9(9):455. doi: 10.3390/biom9090455 (PMC6770808; doi:10.3390/biom9090455)
Supplement: Supplementary file 1 [file biomolecules-09-00455-s001.zip › S2_Table.pdf]

Table S2. Relative mtCN values in the observed tissue samples.

Case No. (ID)	Sample type	Mean mtCN	SD	CV, %
as_01	N	1.00	0.10	9.8
as_01	FI	0.93	-	-
as_01	FS	0.66	0.04	6.5
as_01	FP	0.84	0.09	10.2
as_01	LFP	0.88	0.05	5.6
as_02	N	1.00	0.24	24.3
as_02	FI	0.72	0.10	13.2
as_02	FS	0.91	0.17	18.4
as_02	FP	0.76	0.14	18.7
as_02	LFP	0.60	0.07	12.1
as_02	SM	20.61	-	-
as_02	MM	4.09	-	-
as_02	LIV	3.78	-	-
as_02	SPL	0.68	-	-
as_03	N	1.00	0.01	0.2
as_03	FI	0.94	0.06	6.1
as_03	FS	0.80	0.07	8.5
as_03	FP	0.88	0.05	5.0
as_03	LFP	0.87	0.10	11.8
as_03	SM	21.19	-	-
as_03	MM	5.00	-	-
as_03	LIV	6.89	-	-
as_03	SPL	1.04	-	-
as_04	N	1.00	0.30	29.6
as_04	FI	0.60	0.16	26.9
as_04	FS	0.45	0.00	0.5
as_04	FP	0.75	0.29	38.4
as_04	LFP	0.84	0.21	24.7
as_04	SM	3.79	-	-
as_04	MM	3.40	-	-
as_04	LIV	2.72	-	-
as_04	SPL	0.34	-	-
as_05	N	1.00	0.44	43.8
as_05	FI	0.94	0.41	44.3
as_05	FS	0.63	0.23	36.8
as_05	FP	0.67	0.04	5.7
as_05	LFP	0.67	0.25	37.6
as_05	SM	10.67	-	-
as_05	MM	2.61	-	-
as_05	LIV	4.33	-	-
as_05	SPL	0.77	-	-
as_06	N	1.00	0.05	5.4
as_06	FI	1.04	0.05	4.4
as_06	FS	1.21	0.25	20.5

as_06	FP	1.11	0.20	17.6
as_06	LFP	0.83	0.12	14.6
as_06	SM	5.81	-	-
as_06	MM	10.65	-	-
as_06	LIV	7.91	-	-
as_06	SPL	2.78	-	-
as_07	N	1.00	0.14	13.7
as_07	FI	0.94	0.11	11.5
as_07	FS	0.88	0.08	9.3
as_07	FP	0.95	0.19	19.9
as_07	LFP	1.13	0.06	5.1
as_07	SM	3.78	-	-
as_07	MM	17.15	-	-
as_07	LIV	6.94	-	-
as_07	SPL	0.60	-	-
